# Proteostasis, Assisted Reproductive Technologies, and Neurodevelopmental Differences: An Integrative Perspective

**DOI:** 10.3390/proteomes14020019

**Published:** 2026-04-21

**Authors:** Alberto Fucarino, Yousef Mohamadi, Francesco Cappello, Federica Scalia, Giulia Russo, Giuseppe Gullo, Leila Noori

**Affiliations:** 1 Department of Theoretical and Applied Sciences, eCampus University, 22060 Novedrate, Italy; alberto.fucarino@uniecampus.it; 2 Department of Anatomy, School of Medicine, Ilam University of Medical Sciences, Ilam 6939177143, Iran; yosef.1365@yahoo.com; 3 Department of Biomedicine, Neuroscience and Advanced Diagnostics [BiND], University of Palermo, 90127 Palermo, Italy; francesco.cappello@unipa.it; 4 Department of Medicine and Surgery, Kore University of Enna, 94100 Enna, Italy; federica.scalia@unikore.it; 5 AOOR, Villa Sofia Cervello IVF Unit, University of Palermo, 90100 Palermo, Italy; giuliarusso@gmail.com

**Keywords:** proteostasis, assisted reproductive technologies [ART], neurodevelopmental differences [NDD], molecular chaperones

## Abstract

Proteostasis, defined as the coordinated regulation of protein synthesis, folding, trafficking, and degradation, is essential for maintaining cellular integrity and supporting normal development. During reproduction and early life stages, efficient proteostasis is crucial for gamete quality, successful fertilization, embryonic development, and neurodevelopmental outcomes. Increasing evidence suggests that impaired proteostasis contributes to infertility and may be intertwined with biological vulnerabilities associated with assisted reproductive technologies [ARTs]. This review provides an integrative perspective on the role of disrupted proteostasis in infertility, ART procedures, and neurodevelopmental differences [NDD]. We review epidemiological and molecular findings indicating proteostasis failure in both male and female infertility, with particular emphasis on molecular chaperones. Among these, heat shock protein 60 [Hsp60] is discussed as a central mediator linking mitochondrial function, protein quality control, and reproductive competence. We further highlight that ART procedures coincide with sensitive periods of epigenetic reprogramming and proteostasis regulation during early embryogenesis, indicating that disturbances in proteostasis may affect epigenetic stability and subsequent neurodevelopmental outcomes. In addition, this review emphasizes the importance of proteoforms and proteome complexity as critical determinants of reproductive success and neurodevelopmental robustness in the context of ART. Finally, we discuss the potential of proteomic and chaperone-based biomarkers as emerging tools to optimize ART strategies, improve gamete and embryo selection, and enhance risk assessment and clinical outcomes. The current review underscores proteostasis as a fundamental yet underrecognized mechanism linking reproductive biology, ART outcomes, and long-term neurodevelopment while highlighting future directions for translational investigations.

## 1. Introduction

Proteostasis, the dynamic balance of protein synthesis, folding, and degradation, is a fundamental cellular process required to maintain protein homeostasis and functional integrity [1]. In reproductive biology and early development, efficient proteostasis plays an indispensable role in gametogenesis, fertilization, embryonic development, and the establishment of normal neurodevelopmental trajectories. Disruptions in this finely regulated system may contribute to infertility and adverse developmental outcomes.

In parallel, neurodevelopmental differences [NDDs] have been associated with early-life molecular vulnerabilities, including impaired protein quality control and mitochondrial dysfunction. These processes are particularly sensitive during embryogenesis. Assisted reproductive technologies [ARTs], while highly effective in overcoming infertility, coincide with critical windows of proteostasis regulation and epigenetic reprogramming during early development [2]. In this review, we propose that perturbations in proteostasis may represent a potential mechanistic pathway linking infertility, ART exposure, and neurodevelopmental vulnerabilities, although direct causal evidence remains limited. We synthesize current epidemiological and molecular evidence and discuss how alterations in chaperone activity and proteostasis regulation may influence reproductive success, ART procedures, and neurodevelopmental outcomes.

## 2. Epidemiology of Male and Female Infertility and Protein Homeostasis

Infertility is a major global health concern affecting 15–17% of couples of reproductive age, with increasing prevalence in middle- and high-income regions. The World Health Organization [WHO] defines it as the inability to achieve a clinical pregnancy after 12 months or more of regular unprotected sexual intercourse. Its multifactorial nature, encompassing biological, environmental, and behavioral aspects, impedes epidemiological study, and a substantial proportion of cases remain idiopathic [3]. Approximately 50% of infertility originates from male factors, while the remaining 50% are due to female factors or a combination of both [4].

Proteostasis has recently gained considerable interest as a central mechanism potentially underlying many idiopathic cases. Recognized as fundamental to germ cell function in both sexes, disruption of proteostasis through oxidative stress, aging, metabolic diseases, or mitochondrial dysfunction can impair gamete quality and contribute to infertility [5,6,7].

Proteomic approaches have supported this effect by identifying proteins with altered abundance in association with infertility, correlating epidemiological patterns with underlying biochemical modifications [8,9,10].

Male infertility is often characterized by sperm dysfunction induced by oxidative stress, mitochondrial dysfunction, sperm DNA fragmentation, and protein alterations [8]. Sperm proteomic analyses have revealed significant changes in the abundance of proteins necessary for motility, maturation, and fertilization capacity. Similar changes were also identified in the protein composition of spermatozoa from men with varicocele, a testicular vascular condition associated with hyperthermia and oxidative stress [11]. Mitochondrial uncoupling proteins [UCPs] further demonstrate how altered redox regulation links metabolic disease with impaired sperm function [6], while obesity-driven lipid imbalance and inflammation disrupt sperm proteomes and fertilization competence [5]. Additional evidence indicates apoptotic dysregulation via caspases [12] and proteomic alterations in protein abundance induced by age, smoking, obesity, and pollutants [13]. Notably, sperm proteostasis degradation is also emerging as a systemic health indicator.

In females, proteostasis plays a comparable role in establishing ovarian function, egg quality, and reproductive lifespan. Misfolded protein accumulation decreased degradation capacity, and deficient chaperone activity contributes to diminished oocyte competence and premature ovarian insufficiency [POI] [7,14]. Oocytes rely on stored proteins and tightly regulated proteostatic systems in which disturbances hinder embryonic development [15]. Activation of ER or mitochondrial stress responses can lead to ovarian aging and reduced oocyte quality [4]. Proteomic assessments illustrate that oxidative stress alters the abundance and modification state of key ovarian proteins, impairing maturation and fertilization [10]. Energy metabolism is highly related to proteostasis, as mitochondrial inefficiency, insulin resistance, and metabolic dysfunction minimize the cellular capacity for proper protein synthesis and turnover [15]. Hormonal deregulation can further affect proteostasis via modifying systemic energy homeostasis [16], while low protein diets underscore nutrients affecting mitochondrial integrity and oocyte competence through proteostasis-dependent mechanisms.

Ovarian aging demonstrates the epidemiological impact of proteostasis decline and the deficient clearance of damaged proteins that leads to their aggregation, promoting oocyte senescence and apoptosis and contributing to age-related infertility and poor ART outcomes [4].

Given this, disruptions in proteostasis emerge as a common molecular factor across male and female infertility. Effective germ cell function depends on the chaperone system, the ubiquitin–proteasome system, autophagy, and coordinated metabolic support [7]. These findings may suggest that many idiopathic infertility cases may originate from unrecognized proteostatic failure.

Infertility epidemiology must be understood together with growing environmental, metabolic, and lifestyle risk factors that compromise proteostasis [17,18].

In addition to its roles in gamete biology and embryonic development, disruption of proteostasis has also been implicated in pregnancy-related disorders. Increasing evidence indicates that pre-eclampsia is associated with the accumulation of misfolded and aggregation-prone proteins in the placenta and maternal circulation. Studies have identified amyloid-like protein aggregates, including misfolded transthyretin and other aggregation-prone proteins, in placental tissue and in the urine of women with pre-eclampsia. These aggregates are thought to arise from impaired protein folding capacity and insufficient clearance by cellular quality control systems such as the ubiquitin–proteasome system and autophagy pathways. The resulting proteotoxic stress can contribute to placental dysfunction, oxidative stress, and endothelial damage, key pathological features of pre-eclampsia. These findings indicate the importance of efficient proteostasis networks not only for gamete quality and early embryogenesis but also for maintaining placental function and healthy pregnancy outcomes [19,20].

Taken together, these epidemiological and molecular findings underscore proteostasis as a shared pathway through which diverse reproductive, metabolic, environmental, age-related, and genetic insults converge to impair gamete function [7]. Since disruptions in protein quality control often remain clinically invisible, they may account for a major portion of cases labeled as idiopathic infertility. Furthermore, the same proteostatic vulnerabilities that compromise natural conception likely influence how gametes and embryos respond to the stresses of ART. Recognizing proteostasis failure as a common mechanistic denominator reframes infertility not merely as a hormonal or structural disorder but as a system-level collapse in cellular protein homeostasis [21,22]. This perspective provides a foundation for the next section, which examines, in detail, how the proteostasis machinery ensures gamete quality and early developmental competence.

## 3. The Role of Proteostasis in Gamete Biology

Germ cells are uniquely dependent on proteostasis due to their specialized and metabolically constrained nature. Oocytes can remain arrested in meiosis for decades and are transcriptionally silent for much of their lifespan. As a result, they rely heavily on a finite reserve of stored mRNAs and stable proteins to support subsequent meiotic maturation and early embryogenesis. Spermatozoa are even more specialized, being both transcriptionally and translationally inactive. This makes both cell types particularly vulnerable to proteostatic imbalance, with direct implications for gamete quality, embryo viability, and potentially neurodevelopmental outcomes in the offspring [7,23,24].

A central component of the proteostasis network is the molecular chaperone system. Heat shock proteins [HSPs], among the most well-studied chaperones, facilitate the correct folding of nascent polypeptides, refold misfolded proteins, and direct irreparably damaged proteins toward degradation pathways [25]. This ensures the structural and functional integrity of the germline proteome during the prolonged and stress-prone life cycles of oocytes and spermatozoa [23].

Proteostasis is further supported by selective degradation pathways, including the ubiquitin–proteasome system and autophagy–lysosome pathways. These mechanisms prevent the accumulation of misfolded or aggregated proteins that could otherwise compromise gamete function [26,27]. Recent studies on *Caenorhabditis elegans* have identified a mechanism by which fertilization-associated signals may actively reset proteostasis in the germline. In this model, sperm-derived signals activate lysosomal proton pumps (V-ATPases) in oocytes, leading to lysosomal acidification and enhanced clearance of protein aggregates prior to fertilization. This process restores proteome integrity and may contribute to preparing the embryo for development [28,29]. Importantly, this lysosome-mediated proteostasis renewal occurs before fertilization, indicating that sperm signaling, rather than fertilization itself, may initiate the aggregate-clearance process. Whether similar mechanisms exist in mammalian systems remains unknown.

Although lysosome-mediated clearance may restore proteostasis, an important question is whether incomplete clearance could influence offspring development. Under conditions of high proteotoxic stress, oocyte quality-control systems may be insufficient for eliminating all damaged or misfolded proteins. In such scenarios, residual aggregates or altered protein conformations could persist into early embryogenesis and potentially influence developmental pathways.

Supporting this concept, studies in *Caenorhabditis elegans* have shown that amyloid-like protein assemblies can act as heritable epigenetic elements. These proteoforms can be maintained in the germline and may influence phenotypes across generations. This suggests that protein conformational states may serve as carriers of non-genetic inheritance. Although the relevance of this mechanism to mammalian reproduction remains uncertain, it raises the possibility that proteostasis alterations in germ cells could contribute to intergenerational effects. Further investigation is needed to determine whether similar processes occur in mammalian systems [30].

Germ cells also possess robust oxidative stress response systems, although their capacity varies across developmental stages. Developing germ cells can upregulate antioxidant defenses, whereas mature spermatozoa are particularly vulnerable to oxidative damage. This vulnerability is due to their limited cytoplasm and lack of transcriptional and translational activity, necessitating strict regulation of protein structure and function to maintain cellular integrity. Reactive oxygen species [ROS] can induce oxidative protein modifications, reducing motility and impairing fertilization capacity. Because sperm cannot replace damaged proteins, they rely heavily on existing proteostasis mechanisms [31].

In oocytes, proteostasis capacity declines with age. This decline is associated with the accumulation of oxidized and misfolded proteins and correlates with reduced oocyte quality and fertility. Impaired proteostasis in aging oocytes may also influence mitochondrial integrity, spindle assembly, and chromosomal segregation, processes essential for successful fertilization and embryonic development [32].

Overall, maintenance of proteostasis in gametes, largely mediated by chaperones and protein degradation systems, is critical for reproductive success. Disruption of these processes may not only impair gamete function but could also influence embryonic development and offspring health [33].

Within this network, the mitochondrial chaperonin Hsp60 represents a key regulator of proteome stability in germ cells. Beyond protein folding, Hsp60 contributes to mitochondrial maintenance, steroidogenic regulation, and protection against stress-induced damage. Given its central role at the interface of proteostasis, mitochondrial function, and reproductive competence, Hsp60 provides a useful model for understanding how chaperone dynamics affect fertility [34,35]. The following section thoroughly evaluates its specific roles in the female and male reproductive systems.

## 4. Hsp60, a Significant Chaperone in Gamete Proteostasis and Reproductive Biology

Hsp60 is a mitochondrial chaperonin that plays a central role in maintaining mitochondrial protein folding, assembly, and functional stability of mitochondrial proteins. It belongs to the chaperonin family of molecular chaperones and forms large oligomeric complexes within the mitochondrial matrix. Structurally, Hsp60 assembles into double-ring structures composed of multiple subunits that create a central folding chamber in which unfolded polypeptides can undergo ATP-dependent refolding. This activity occurs in cooperation with its co-chaperone Hsp10, which acts as a lid-like structure regulating substrate encapsulation and release. The activity of this complex is dynamically regulated by cellular stress responses and post-translational modifications, which influence its localization, stability, and substrate interactions. These regulatory mechanisms contribute to the maintenance of mitochondrial proteostasis under both physiological and stress conditions [36]. Consistent with its central role in mitochondrial homeostasis, Hsp60 participates in multiple processes across the reproductive axis, including oogenesis, folliculogenesis, spermatogenesis, steroidogenesis, implantation, and early embryonic development. Emerging evidence further indicates that Hsp60 contributes to mitochondrial stress adaptation and immune-mediated reproductive dysfunction, features that distinguish it from several other molecular chaperones. Proper mitochondrial proteostasis is critical for reproductive competence, as mitochondria provide the energy, metabolic regulation, and apoptosis signaling required for germ cell maturation and early embryonic development. Reflecting this central role, Hsp60 has emerged as an important regulator at the interface of mitochondrial function, cellular stress responses, and protein quality control, making it a highly relevant candidate for investigation in reproductive biology [37]. Owing to this combination of mitochondrial specificity and reproductive relevance, Hsp60 provides an informative framework for exploring mechanisms of infertility and potential vulnerabilities associated with ART. The following subsections examine its sex-specific roles, molecular mechanisms, and contributions to gamete quality and reproductive competence.

### 4.1. Hsp60 in the Female Reproductive System

Hsp60 is present in oocytes throughout follicular growth and possibly contributes to reproductive maturation and hormonal production [35]. Altered Hsp60 abundance and localization have been observed during ovarian pathology, like cystic ovarian disease, where Hsp60 is overexpressed due to inflammation, while it is less expressed in luteal insufficiency associated with decreased progesterone synthesis [38,39]. In Drosophila, Hsp60C abundance varies throughout oogenesis, particularly during cytoskeletal reorganization, which is critical for proper oocyte development [40]. Further, Hsp60 is present in the bovine oviduct epithelium, which may regulate sperm binding and gamete transport and adjust the microenvironment to support fertilization and embryo development [41]. Hsp60 abundance also changes through the menstrual cycle in endometrial cells, increasing during proliferative and early secretory phases and reinforcing implantation [42,43]. Uteric and embryonic Hsp60 levels are diminished during inflammatory reactions, leading to disrupted implantation and early pregnancy maintenance in mice [44]. Similar fluctuations occur in sheep during early stages of pregnancy [45]. Moreover, surface localization of Hsp60 in uterine and oviductal epithelial cells can affect sperm motility. Promoting general sperm movement, it may counteract IBMX-induced hyperactivation, underscoring its context-dependent role [46].

### 4.2. Hsp60 in the Male Reproductive System

Hsp60 is detected in multiple male reproductive cell types, including Leydig cells, Sertoli cells, spermatogonia, and early spermatocytes. It is also involved in steroidogenesis and germ cell maturation [47,48,49]. During heat stress, increased Hsp60 abundance has been reported in Sertoli cells and spermatogonia, preventing apoptotic damage to the seminiferous epithelium and improving germ cell survival [50,51]. In infertile men, compromised Hsp60 immunoreactivity is associated with deficient spermatogenesis due to disrupted mitochondrial homeostasis essential for germ cell proliferation [52,53]. Seminal frequent exhibition of Hsp60 and anti-Hsp60 antibodies, along with anti-sperm antibodies, has been observed in men with chronic genital tract infections, linking Hsp60 to immune-related male infertility [54]. Moreover, Hsp60 shows increased abundance in testes over exposure to environmental stresses, indicating its cytoprotective and anti-apoptotic importance [55,56]. Hsp60 deficiency is associated with genetic variants of mitochondrial assembly in sperm and drastic asthenoteratozoospermia [57,58]. Hsp60 abundance also decreases due to aging in senile rabbits, leading to impaired spermatogenic activity [59]. These findings highlight the potential contribution of chaperonopathies in male infertility. Of note, Hsp60 distribution varies over the sperm journey through the epididymis, with a lowering in the tail that rises in the acrosomal region, pointing toward a role in zona pellucida recognition and capacitation [60,61]. However, Hsp60 remains in the midpiece of ejaculated sperms, indicating its mitochondrial association [62]. It also offers noncanonical extracellular roles related to fertilization, localizing on the sperm surface to interact with the oviductal epithelium [63].

## 5. Hsp60 as Biomarker for ART and Reproductive Health

Hsp60, a central member of the chaperone system, may be assumed to be a promising biomarker in infertility and ART improvement. Higher levels of Hsp60 have been seen in impaired semen parameters and immune-related infertility. In females, variations in Hsp60 abundance in the ovary, oviduct, or endometrium may indicate compromised folliculogenesis or implantation competence. Chlamydia trachomatis expresses a homologous Hsp60 [CHsp60], stimulating solid immune reactions. Owing to structural mimicry with human Hsp60, anti-CHsp60 antibodies exhibit antigenic overlap with reproductive tissues, contributing to inflammation and tubal infertility [64,65,66,67,68,69,70]. In the assisted reproduction context, serum-positive anti-CHsp60 is correlated with declined embryo implantation rates and hindered endometrial receptivity, endorsing its clinical relevance [71,72]. Diagnostic protocols of tubal-related infertility are increasingly integrating measurements of anti-Hsp60 antibodies as predictive biomarkers [73,74,75,76]. In experimental and clinical studies, Hsp60 expression has been evaluated using immunological and molecular techniques, including immunofluorescence and immunohistochemistry on paraffin-embedded tissue sections, enzyme-linked immunosorbent assays (ELISAs), Western blotting for protein detection, and PCR-based approaches or in situ hybridization to assess gene expression and spatial localization. These measurements have been performed in various biological samples relevant to reproductive biology, including plasma, follicular fluid, germ cells, and reproductive tissues. Given this, Hsp60 assessment during ART may enhance patient fulfillment and implantation outcomes.

## 6. ART and Neurodevelopmental Risks

The relationship between ART, proteostasis, and neurodevelopment remains incompletely understood. The following section, therefore, presents the available evidence within a hypothesis-generating framework rather than implying a direct causal relationship. ART procedures, such as in vitro fertilization [IVF] and intracytoplasmic sperm injection [ICSI], have revolutionized infertility treatment, enabling countless individuals to achieve parenthood [77]. As the number of ART-conceived births continues to rise worldwide, increasing attention has been directed toward the long-term health and developmental outcomes of these individuals. Several studies have examined associations between ART and neurodevelopmental outcomes, including outcomes such as autism spectrum differences [ASD], intellectual disability, and cerebral palsy. While some reports suggest modest increases in the prevalence of certain neurodevelopmental conditions, particularly following procedures such as ICSI, the underlying biological mechanisms, if any, remain unclear [78]. Importantly, many studies emphasize that these associations are frequently influenced by confounding factors such as parental infertility, advanced parental age, genetic background, prematurity, and multiple pregnancies, all of which occur more frequently in ART populations [79].

More broadly, epidemiological studies assessing neurodevelopmental outcomes in ART-conceived children have produced mixed findings. Some analyses report modest increases in the diagnosis of certain neurodevelopmental conditions; however, these associations are often attenuated after adjustment for confounding factors such as prematurity, low birth weight, parental age, and underlying infertility. Comparative studies examining siblings conceived with and without ART frequently report largely comparable cognitive, behavioral, and psychomotor development [77]. Collectively, these observations suggest that many reported differences are likely attributable to underlying parental or perinatal factors rather than the ART procedures themselves.

Overall, current evidence does not support a clear causal relationship between ART procedures and neurodevelopmental differences, although a small increase in risk cannot be entirely excluded. Continued long-term follow-up studies remain important for clarifying these associations. Given these uncertainties, increasing research interest has focused on identifying biological mechanisms that are proposed to modulate early developmental programming during ART. Processes such as proteostasis, mitochondrial function, and epigenetic regulation have been proposed as potential contributors to early embryonic homeostasis. While these mechanisms are known to play essential roles during embryogenesis, their possible contribution to long-term developmental variability remains largely hypothetical and requires further investigation [77]. Table 1 summarizes ART procedures and reported associations with neurodevelopmental outcomes.

## 7. Proteostasis and Epigenetics: Interconnected Mechanisms in ART and Development

**Figure 1:**
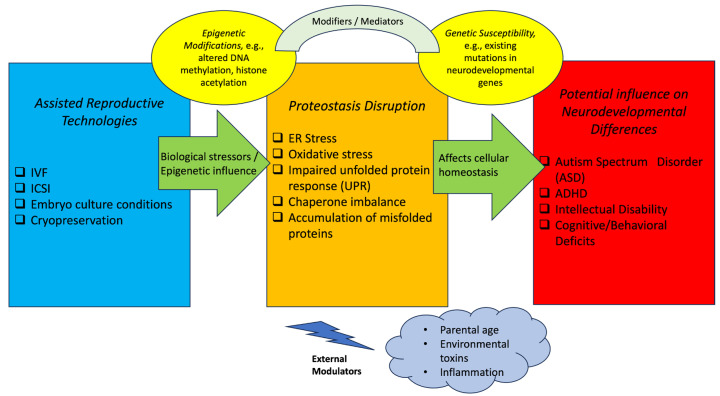
Conceptual framework illustrating potential mechanistic pathways linking ART-related proteostasis disruption to neurodevelopmental differences. This figure presents a hypothesis-generating model based on emerging evidence from reproductive biology, proteomics, and developmental neuroscience. Solid lines indicate established biological processes. Confounding factors include parental age, infertility etiology, and perinatal complications. Further longitudinal studies and mechanistic investigations are needed to establish causality.

Emerging evidence suggests that epigenetic mechanisms, particularly alterations in DNA methylation patterns, may contribute to developmental variabilities observed following ART. As ART procedures coincide with critical windows of epigenetic reprogramming during early embryogenesis, environmental and procedural factors associated with in vitro manipulation have the potential to influence gene expression programs involved in early neurodevelopment [85]. Importantly, these epigenetic processes do not operate in isolation but are closely intertwined with broader cellular homeostasis systems, including proteostasis. While the epigenetic consequences of ART have been extensively studied, the potential contribution of protein quality control systems and molecular chaperones remains comparatively underexplored. Therefore, an integrated view in which epigenetic regulation and proteostasis interact may be important for shaping embryonic developmental patterns (Figure 1) [86,87,88].

Under the DOHaD framework, prenatal factors such as maternal immune activation, malnutrition, psychological stress, and environmental exposures may influence neurodevelopment by affecting pathways related to neurogenesis, synapse formation, and neuronal connectivity [89]. Many of these stressors are also known to simultaneously affect the proteostasis network [90,91]. For instance, molecular chaperones and stress-response proteins can modulate the stability and activity of epigenetic regulators, including DNA methyltransferases, histone-modifying enzymes, and chromatin-associated proteins. Heat shock proteins such as Hsp90 have been shown to stabilize key chromatin regulators, including DNMT1, histone methyltransferase EZH2 of the PRC2 complex, and histone deacetylases such as HDAC1 and HDAC6, thereby protecting them from proteasomal degradation and supporting the maintenance of DNA methylation patterns. Similarly, the ubiquitin–proteasome system regulates the turnover of epigenetic modifiers, contributing to chromatin dynamics during development [92]. These observations suggest a functional interplay between proteostasis and epigenetic regulation, although the precise mechanisms remain incompletely understood [93,94].

Proteostasis-related mechanisms are thought to contribute to cellular resilience during embryogenesis. Late embryogenesis abundant [LEA] proteins such as HeLEA1 from tardigrades preserve membrane integrity under stress, acting as chaperones that stabilize mitochondrial membranes during developmental stress [95]. These functions may be particularly relevant in the ART context, where embryos are exposed to oxidative, thermal, and osmotic stressors that may affect both protein homeostasis and epigenetic stability. The extent to which these factors influence long-term developmental outcomes remains to be clarified [95,96].

Protein aggregation, once considered primarily pathological, is now recognized as a regulated process involved in developmental control. Biomolecular condensates formed through dynamic protein interactions are known to influence gene expression, chromatin organization, and spatial regulation of developmental processes. Molecular chaperones ensure proper assembly and disassembly of these condensates, further supporting a potential link between proteostasis and epigenetic regulation during early development [96,97].

Molecular chaperones are known to play important roles in neural development. HSPs, such as Hsp70 and Hsp90, are involved in signaling pathways regulating neuronal proliferation, migration, and differentiation, while endoplasmic reticulum chaperones, including BiP, calnexin, and calreticulin, contribute to synaptic protein folding and maturation [98,99]. Moreover, mitochondrial proteostasis has been implicated in neuronal development, with disruptions affecting synapse formation and neuronal signaling [100]. These findings underscore the importance of protein quality control mechanisms in the developing nervous system, although their potential links to ART-related developmental variability remain largely hypothetical.

Additional pathways, including the mitochondrial unfolded protein response (UPRmt) and autophagy, further support cellular quality control during development. These processes are involved in maintaining proteostasis under stress conditions and have been associated with neurodevelopmental processes, although their specific roles in the ART context remain to be fully elucidated [100].

Taken together, proteostasis and epigenetic regulation appear to be interconnected components of early developmental biology. However, current evidence primarily supports an associative rather than a causal relationship between proteostasis alterations and neurodevelopmental outcomes. These processes are best understood within a multifactorial framework involving environmental influences, cellular stress responses, and epigenetic regulation.

## 8. Chaperones as Integrative Hubs in Cellular Regulation

Molecular chaperones act as central integrative hubs across multiple cellular pathways essential for embryogenesis and neurodevelopment. Beyond their canonical role in protein folding, chaperones coordinate diverse processes, including protein degradation, mitochondrial function, stress responses, and metabolic regulation [101,102,103]. As discussed in Section 7, chaperone systems also interact with epigenetic regulators; however, their broader role as system-level coordinators extends across multiple aspects of cellular homeostasis. Chaperones contribute to mitochondrial integrity, including mtDNA stability, respiratory function, and activation of the mitochondrial unfolded protein response [UPRmt], which are crucial for oocyte competence and early neuronal differentiation [104,105,106]. Hsps also buffer oxidative stress by stabilizing antioxidant pathways and limiting damage induced by ROS [107]. Moreover, chaperones interface with metabolic signaling pathways such as AMPK and mTOR, linking cellular energetics with protein quality control and developmental progression [108,109,110]. Collectively, these overlapping functions position chaperones as master regulators of cellular resilience and developmental robustness. Their central role across interconnected pathways suggests that disturbances in chaperone activity, whether arising from intrinsic factors or ART-related stressors, may influence developmental processes at multiple levels [111,112]. This integrative perspective supports their potential as biomarkers and therapeutic targets in reproductive medicine.

## 9. Proteoform Complexity and Developmental Proteostasis

Modern proteomics demonstrates that biological function is shaped not only by total protein abundance but also by proteoform diversity arising from alternative splicing, post-translational modifications, proteolytic processing, and conformational variation [113]. During gametogenesis and early embryogenesis, proteome complexity is notably high, indicating rapid cellular transitions, plastic metabolic demands, and extensive epigenetic remodeling [114,115].

The proteostasis network does not solely supervise protein quantity but also supervises the balance, stability, localization, and functionality of proteoforms. Disruption in chaperone systems, proteasomal degradation, or autophagy may then lead to modest yet biologically significant changes in proteoform composition rather than global protein loss [116,117]. As outlined in Section 7, such changes may intersect with epigenetic regulations and developmental processes.

In the context of ART, non-physiological environment exposure may affect proteoform equilibrium via oxidative stress, altered metabolic flux, and temperature and osmotic variations in developing embryos. Such stressors may alter the embryonic proteome, promoting maladaptive proteoforms, impairing protein–protein interactions, and destabilizing biomolecular condensates crucial for epigenetic regulation. Importantly, neurodevelopmental vulnerabilities may also emerge from persistent imbalance in proteoform networks and not always from gross proteomic defects [118]. This perspective can mechanistically link ART-associated environmental stress, disrupted proteostasis, and long-term neurodevelopmental outcomes.

## 10. Clinical Translation: Biomarkers and ART Optimization

The growing understanding of proteostasis in reproductive biology has prompted increasing interest in its clinical translation, particularly in the identification of biomarkers for assessing gamete and embryo quality and optimizing ART outcomes. Proteomic analyses have highlighted molecular chaperones, especially HSPs, as promising candidates in this context [119]. HSPs serve as crucial protectors of gametes during manipulation and early embryogenesis. Their abundance has been associated with improved fertilization rates and embryo viability, marking them as key targets for ART enhancement [120]. Balanced HSP abundance supports oocyte maturation and sperm functionality. Conversely, altered HSP abundance has been linked to infertility and compromised embryo development [33]. These findings position HSP profiling as a promising precision-medicine tool to guide embryo selection, optimize laboratory conditions, and stratify patients according to underlying proteostatic susceptibility. In practical ART settings, chaperone profiling could be incorporated into semen analysis through quantification of HSPs such as HSPA2, Hsp70, Hsp90, and Hsp60 using various molecular techniques, including ELISA and Western blotting, alongside immunofluorescence assessments of chaperone localization, particularly HSPA2 at the equatorial segment, which predicts fertilization competence [121]. Emerging microfluid assays may further enable fast detection of surface chaperones for motile sperm sorting. In parallel, oocyte quality could be examined through cumulus cell RNA/protein profiling of HSPs and by applying secretome proteomics to spent culture media, while embryo-conditioned media could be monitored for HSPs release as indicators of developmental stress or resilience. However, implementation remains constrained by the cost and expertise required for mass spectrometry, limited assay standardization, and the need for harmonized reference ranges and validated clinical thresholds [122,123]. Regulatory approval will further require proof that chaperone biomarkers offer predictive value beyond conventional morphology- and kinetic-based analyses. Accordingly, large cohorts are required to define concentration and localization ranges for key chaperones [HSPA2, Hsp70, Hsp90, and Hsp60] in fertile versus infertile populations. Studies must evaluate sensitivity, specificity, and likelihood ratios for predicting outcomes such as fertilization rate, blastocyst development, implantation rate, and live birth [60,124]. Preliminary data suggest HSPA2 deficiency strongly predicts poor IVF fertilization, with sensitivity estimates in some reports, although systematic validation is lacking. Diminished HSPA2 is a hallmark of defective sperm maturation and is highly associated with impaired zona pellucida binding and failed fertilization in conventional IVF [125]. Given that ICSI can bypass the need for sperm–oocyte recognition, it can circumvent HSPA2-related defects, enabling fertilization even when sperm maturation is not optimal. Finally, randomized trials would test whether HSP-guided decision-making, like choosing ICSI for men with low HSPA2, can improve ART outcomes [121,126]. However, if HSPA2 deficiency reflects broader proteostasis failure, including DNA repair defect or altered paternal epigenetic marks, ICSI may fertilize an oocyte using sperm that nature would have excluded [125]. Of note, HSPA2 deficiency is likely associated with broader spermatogenic dysfunction, including increased oxidative damage, abnormal chromatin remodeling, and altered epigenetic marking [111,127]. This connects closely with the considerations surrounding ART-associated NDD risk, as suboptimal sperm proteostasis may contribute to paternal-related susceptibility in certain neurodevelopmental pathways. Although most studies report no major increase in global NDD in ICSI-conceived children [128,129,130], some large cohort analyses have identified a modest but statistically significant association between ICSI and conditions such as autism spectrum disorders or developmental delay, particularly when male-factor infertility is severe. Thus, it indicates the need for meticulous elaboration on whether bypassing sperm quality during ICSI causes downstream developmental consequences and careful interpretation of ICSI’s protective role [21,131].

Chaperone-based sperm selection has attracted increasing interest as an alternative to morphology- or motility-related methods. Surface HSPs serve as markers of sperm maturity and fertilizing potential, with disruptions linked to male infertility. Emerging technologies such as microfluidic sorting, immunoaffinity selection, or label-free spectroscopic approaches may provide for sperm selection with intact proteostasis [132].

The chaperone system represents a mechanistically grounded and clinically relevant biomarker class that bridges molecular pathology with ART outcomes. Their profiling offers insights that traditional semen analysis or oocyte morphology cannot provide. However, technical and validation barriers remain; integrating chaperone assessment into the ART workflow may be promising for refining patient classification, assisting in procedural selection, and enhancing reproductive success [120]. Table 2 summarizes some key biomarkers and their roles in ART optimization.

## 11. Conclusions and Future Directions

This review goes beyond summarizing the existing association between infertility, ART, and neurodevelopmental outcomes by proposing proteostasis and proteoform-level regulation as a unifying mechanistic framework. By integrating evidence from reproductive biology, proteomics, epigenetics, and neurodevelopment, we highlight previously unacknowledged molecular vulnerabilities introduced during ART procedures. This system-level perspective identifies actionable gaps in current research and clinical practice and provides a roadmap for advancing both mechanistic understanding and translational applications in reproductive medicine.

Proteostasis emerges as a critical yet underappreciated mediator at the intersection of ART and neurodevelopment. In this review, we emphasized how ART procedures coincide with the vulnerable window of epigenetic reprogramming and proteostatic regulation during early embryogenesis. While epigenetic alteration, particularly in DNA methylation, has dominated current discourse on ART-associated neurodevelopmental risk, converging evidence indicates that proteostasis, including molecular chaperones, UPR and UPRmt, autophagy, and stress-response pathways, plays a parallel and deeply interconnected role in shaping developmental outcomes. Key insights from recent studies suggest that cytoskeletal organization, mitochondrial integrity, biomolecular condensates, and protein folding networks are all influenced by ART-related environmental conditions, with possible long-term consequences for neurogenesis, synaptic maturation, and neuronal connectivity.

Importantly, advancing beyond bulk proteomic approaches, proteoform-resolved analyses offer a more refined understanding of these processes. Applied to gametes and early embryos, such approaches may reveal how specific proteoforms of molecular chaperones, stress-response proteins, and epigenetic regulators are altered during ART procedures or under conditions of proteostatic stress. These analyses can uncover subtle changes in protein folding states, aggregation-prone species, and post-translationally modified variants that are not detectable using standard proteomic techniques. Ultimately, this level of resolution may improve our understanding of how proteostasis networks operate in reproductive cells and may facilitate the identification of proteostasis-based biomarkers associated with gamete quality and embryo viability.

From a clinical perspective, this integrated view of epigenetics and proteostasis broadens our understanding of why some ART-conceived individuals may show subtle increases in neurodevelopmental vulnerabilities while the majority remain within typical developmental trajectories. Importantly, these mechanisms also align with the DOHaD framework, positioning ART not as a standalone risk factor but as part of a broader landscape of prenatal influences, including maternal health, metabolic status, inflammation, and environmental exposures, that collectively shape neurodevelopmental resilience or susceptibility. Recognizing the role of proteostasis may ultimately support the refinement of ART laboratory conditions, embryo condition media, oxygen tension management, and cryopreservation protocols to minimize developmental stress.

Importantly, it should be emphasized that the vast majority of children conceived through ART develop within typical cognitive, behavioral, and neurological ranges. While epidemiological studies have identified modest increases in certain neurodevelopmental risks, these associations are generally small in magnitude and often influenced by underlying parental, genetic, or environmental factors. Therefore, ART should not be viewed as a direct cause of adverse neurodevelopmental outcomes but rather as one component within a multifactorial developmental context. Notably, the benefits of ART in enabling parenthood for individuals with infertility remain substantial, and current evidence supports its overall safety. A balanced interpretation of risk is essential to avoid overestimation of potential adverse outcomes while continuing to refine clinical practice.

Despite growing interest, major knowledge gaps remain. The field still lacks comprehensive datasets linking proteostatic alterations in early embryos to later neurodevelopmental outcomes in humans. Mechanistic studies have not fully resolved how specific ART interventions modify chaperone networks, stress dynamics, mitochondrial proteome stability, or the post-translational modification landscape. Moreover, no studies to date have integrated multi-omics datasets, combining proteomics, epigenomics, metabolomics, and single-cell transcriptomics, to capture the full interplay between proteostasis and epigenetic programming in ART embryos or ART-conceived children. Current data are insufficient to establish a direct causal link or clarify whether proteostasis alteration during assisted reproduction contributes to developmental programming in offspring.

Future research should focus on integrating proteostasis into ART research through targeted and scalable approaches. Priorities include the application of proteoform-resolved proteomics and single-cell multi-omics to early embryos, as well as longitudinal cohort studies linking early molecular signatures to neurodevelopmental outcomes. Mechanistic models, including advanced in vitro embryo systems, organoids, and animal models, are needed to clarify how ART conditions influence chaperone networks and proteome stability. In parallel, the development of standardized proteostasis-based biomarkers may support improved embryo selection, risk stratification, and personalized ART strategies. Collectively, these efforts will be essential to translate proteostasis insights into clinical practice and improve long-term developmental outcomes.

## Figures and Tables

**Table 1:** ART procedures and associated neurodevelopmental risks.

ART Procedure	Associated Neurodevelopmental Risks	Key Findings and Notes	Ref.
In vitro fertilization [IVF]	Slightly elevated risks for learning and motor differences [in singletons]	No substantial risk difference compared to natural conception; no difference between frozen vs. fresh embryo transfer	[80]
Intracytoplasmic sperm injection [ICSI]	Elevated risk of ASD and developmental delay	Increased risk specifically associated with ICSI, independent of parental infertility	[79]
ART without ICSI	No significant increase in NDD risk	Supports the notion that ICSI, not ART in general, may drive neurodevelopmental vulnerability	[78]
General ART-conceived children	Increased diagnoses of ASD, intellectual disability, and cerebral palsy [modest]	Risks may be mediated by preterm birth, low birth weight, and higher medical surveillance rates	[81]
General ART [meta-analysis]	↑ CP, ID, ASD; especially with ICSI; no difference frozen vs. fresh embryo	CP: RR 1.82; ICSI increases ID [RR 1.46] and ASD [RR 1.49]; preterm birth [RR 2.22] and LBW [RR 1.80] are mediators	[78]
ART (longitudinal birth cohort)	Mostly reassuring neurodevelopmental outcomes in singletons; possible risk in gross motor development	ART singletons show lower risk of deficits in cognition and communication domains but higher risk of gross motor delay	[82]
ART-conceived children (perinatal outcome study)	Neurodevelopmental vulnerability associated with brain immaturity	Perinatal factors (prematurity, low birth weight, and neonatal morbidity) contribute later neurodevelopmental differences; early brain immaturity is a key factor	[83]
General ART outcomes (review)	Increased risk of cerebral palsy and epilepsy; possible increase in ADHD and behavioral differences: autism data inconclusive	Many risks are mediated by prematurity, low birth weight, and multiple births; some may reflect parental subfertility rather than ART itself; with possible imprinting errors	[84]

**Table 2:** Key biomarkers and their roles in ART optimization.

Biomarker/Chaperone	Role in Gamete/ Embryo Quality	Normal/ Expected Range *	Measurement Methods	Clinical/ART Implication	Strength of Evidence	Ref.
Heat shock proteins [HSPs]	Protect gametes during manipulation and early embryogenesis	No universal defined ranges, increased Hsp70/Hsp90 leads to better stress tolerance	Western blot, ELISA, secretome proteomics	Expression correlates with improved fertilization rates and embryo viability	Moderate [multiple proteomics studies, limited clinical translation]	[119]
HSPA2	Supports sperm maturation and fertilization capacity	Fertile men typically show high equatorial segment expression; infertile men often show marked reduction	Immunofluorescence, ELISA, flow cytometry	Low levels impair fertilization, particularly in conventional IVF; ICSI can bypass this deficit	Strong [consistently replicated in human sperm studies]	[121]
Chaperones [general]	Biomarkers for sperm quality and selection	No standardized cut-offs; qualitative patterns as disrupted localization	Proteomics, immunostaining, microfluidic sperm sorting	Sperm selection techniques using chaperone profiles may improve ART success	Moderate [growing evidence from functional and proteomic studies]	[132]
Late embryogenesis abundant [LEA] proteins [e.g., HeLEA1]	Stabilize membranes during developmental stress	Not yet clinically characterized	Experimental proteomic assays	Potential protective factor during embryo manipulation or stress conditions	Preliminary [early translational research only]	[95]

* Normal/Expected Range are not standardized, and may vary across studies, populations, and measurement methods.

## Data Availability

No data were created or analyzed in this study. All information discussed is derived from previously published studies, which are cited within the article.

## Abbreviations

The following abbreviations are used in this manuscript:

AMPK	AMP-activated protein kinase;
ARTs	Assisted reproductive technologies;
ASD	Autism spectrum disorder;
BiP	Binding immunoglobulin protein;
CHsp60	Chlamydia heat shock protein 60;
DOHaD	Developmental origins of health and disease;
ELISA	Enzyme-linked immunosorbent assay;
HSP	Heat shock protein;
ICSI	Intracytoplasmic sperm injection;
IVF	In vitro fertilization;
LEA	Late embryogenesis abundant;
mTOR	Mechanistic target of rapamycin;
NDD	Neurodevelopmental differences;
POI	Premature ovarian insufficiency;
ROS	Reactive oxygen species;
UPR	Unfolded protein response;
UCP	Uncoupling protein;
WHO	World health organization.
